# Neuroprotective Effect of Gold Nanoparticles and Alpha-Lipoic Acid Mixture against Radiation-Induced Brain Damage in Rats

**DOI:** 10.3390/ijms23179640

**Published:** 2022-08-25

**Authors:** Noha F. Abdelkader, Ahmed I. El-Batal, Yara M. Amin, Asrar M. Hawas, Seham H. M. Hassan, Nihad I. Eid

**Affiliations:** 1 Department of Pharmacology and Toxicology, Faculty of Pharmacy, Cairo University, Kasr El-Aini Street, Cairo 11562, Egypt; 2 Department of Drug Radiation Research, National Centre for Radiation Research and Technology (NCRRT)—Egyptian Atomic Energy Authority, Cairo 11787, Egypt

**Keywords:** radiation, brain damage, gold nanoparticles, alpha-lipoic acid, oxidative stress, DNA fragmentation

## Abstract

The current study aims to evaluate the possible neuroprotective impact of gold nanoparticles (AuNPs) and an alpha-lipoic acid (ALA) mixture against brain damage in irradiated rats. AuNPs were synthesized and characterized using different techniques. Then, a preliminary investigation was carried out to determine the neuroprotective dose of AuNPs, where three single doses (500, 1000, and 1500 µg/kg) were orally administrated to male Wistar rats, one hour before being exposed to a single dose of 7Gy gamma radiation. One day following irradiation, the estimation of oxidative stress biomarkers (malondialdehyde, MDA; glutathione peroxidase, GPX), DNA fragmentation, and histopathological alterations were performed in brain cortical and hippocampal tissues in both normal and irradiated rats. The chosen neuroprotective dose of AuNPs (1000 µg/kg) was processed with ALA (100 mg/kg) to prepare the AuNPs-ALA mixture. The acute neuroprotective effect of AuNPs-ALA in irradiated rats was determined against valproic acid as a neuroprotective centrally acting reference drug. All drugs were orally administered one hour before the 7Gy-gamma irradiation. One day following irradiation, animals were sacrificed and exposed to examinations such as those of the preliminary experiment. Administration of AuNPs, ALA, and AuNPs-ALA mixture before irradiation significantly attenuated the radiation-induced oxidative stress through amelioration of MDA content and GPX activity along with alleviating DNA fragmentation and histopathological changes in both cortical and hippocampal tissues. Notably, the AuNPs-ALA mixture showed superior effect compared to that of AuNPs or ALA alone, as it mitigated oxidative stress, DNA damage, and histopathological injury collectively. Administration of AuNPs-ALA resulted in normalized MDA content, increased GPX activity, restored DNA content in the cortex and hippocampus besides only mild histopathological changes. The present data suggest that the AuNPs-ALA mixture may be considered a potential candidate for alleviating radiation-associated brain toxicity.

## 1. Introduction

For many years, radiation therapy has been the standard therapy for brain tumors [1,2]. It either kills cancer cells directly or prevents them from growing. Acute or chronic adverse effects may occur in patients receiving brain irradiation. In the central nervous system, ionizing radiation causes the generation of intracellular reactive oxygen species (ROS) and pro-inflammatory cytokines, resulting in brain damage [3]. Several studies proved the role of oxidative stress and inflammation as substantial pathways leading to radiation-induced brain injury [4,5]. In addition, radiation destroys DNA directly via its ionization and indirectly via radioactive free radicals which react with DNA leading to its damage [6]. It is widely known that radiation-induced genomic mutations may be related to DNA damage. Thus, heavy atom radiosensitizers were utilized as effective dose enhancers during radiotherapy for tumor treatment, to maximize the dose delivery for tumor tissues while keeping it minimal at surrounding normal tissues. Recently, it has been reported that gold nanoparticles (AuNPs) were effective radiosensitizers. The efficacy of AuNPs in proton radiation was affected by the nanoparticles’ size, proton beam energy, and the distance between the nanoparticle and DNA. The maximum dose enhancement ratio was achieved with a 25 nm AuNPs that were 30 nm away from the DNA and were irradiated by a 0.5 MeV proton beam [7]. Moreover, Jabeen and Chow [8] demonstrated that the magnetic field increased the dose enhancement ratio for AuNPs when used as radiosensitizers in radiotherapy.

Nanoparticles (NPs) are one of the most promising and adaptable medication delivery technologies for difficult-to-reach areas such as the brain. In neurodegenerative/ischemic illnesses, nanoparticles can deliver therapeutic medicines into damaged areas of the brain by improving their transport via the blood–brain barrier (BBB), as well as targeting important brain regions for regenerative processes [9].

Gold is a rare element that has been utilized as anti-HIV, antiangiogenetic, anti-malarial, and anti-arthritic [10,11,12]. Because of the biocompatibility and robust interaction of gold with soft bases such as thiols, it plays a key role in cancer treatment [13]. Furthermore, prior studies have explained that AuNPs have potential antioxidant effects [14] and are efficient in the dose-dependent tempering of ROS [15]. Indeed, Because of its excellent inherent features, including strong chemical stability, surface functionalization, and well-controlled size, AuNPs have been extensively researched with promising results for its application in drug delivery, gene therapy, photothermal therapy, radiotherapy, and diagnostic purposes [16,17]. Metallic nanoparticles can be made in various means, the most popular of which is the citrate reduction chemical technique [17].

Alpha-lipoic acid (ALA), a disulfide derivative of octanoic acid, is very well recognized for its antioxidant, anti-inflammatory, and anticancer activities. Moreover, ALA is often used to treat neurological illnesses such as diabetic polyneuropathy and multiple sclerosis. Recycling, direct radical scavenging, metal chelation, endogenous antioxidants’ regeneration, and regulation of the expression of various antioxidants and anti-inflammatory genes are all examples of its antioxidant impact. The five-membered ring’s stretched conformation in the intramolecular disulfide confers antioxidant and radioprotective capabilities to ALA, which explains its relatively excellent scavenging activity [18].

Valproic acid (VPA) has been broadly utilized as an antiepileptic agent [19]. VPA is used in the current study as a reference central neuroprotective drug [20]. VPA impacts neuronal proliferation/differentiation stability, survival/apoptosis, and synaptic plasticity by acting directly on neurons as well as indirect means via glial cells [19].

The aim of this study is to evaluate whether an AuNPs mixture with ALA could protect the brain of irradiated rats against radiation-induced damage. Firstly, AuNPs were synthesized and characterized using different techniques. Then, a preliminary study was done to determine the neuroprotective dose of AuNPs followed by evaluation the acute neuroprotective effect of the AuNPs-ALA mixture in irradiated rat brain tissues, namely, cortex and hippocampus, against VPA as a CNS neuroprotective standard drug.

## 2. Results

### 2.1. Characterization of Gold Nanoparticles

#### 2.1.1. Ultraviolet/Visible Spectroscopy

**Figure 1:**
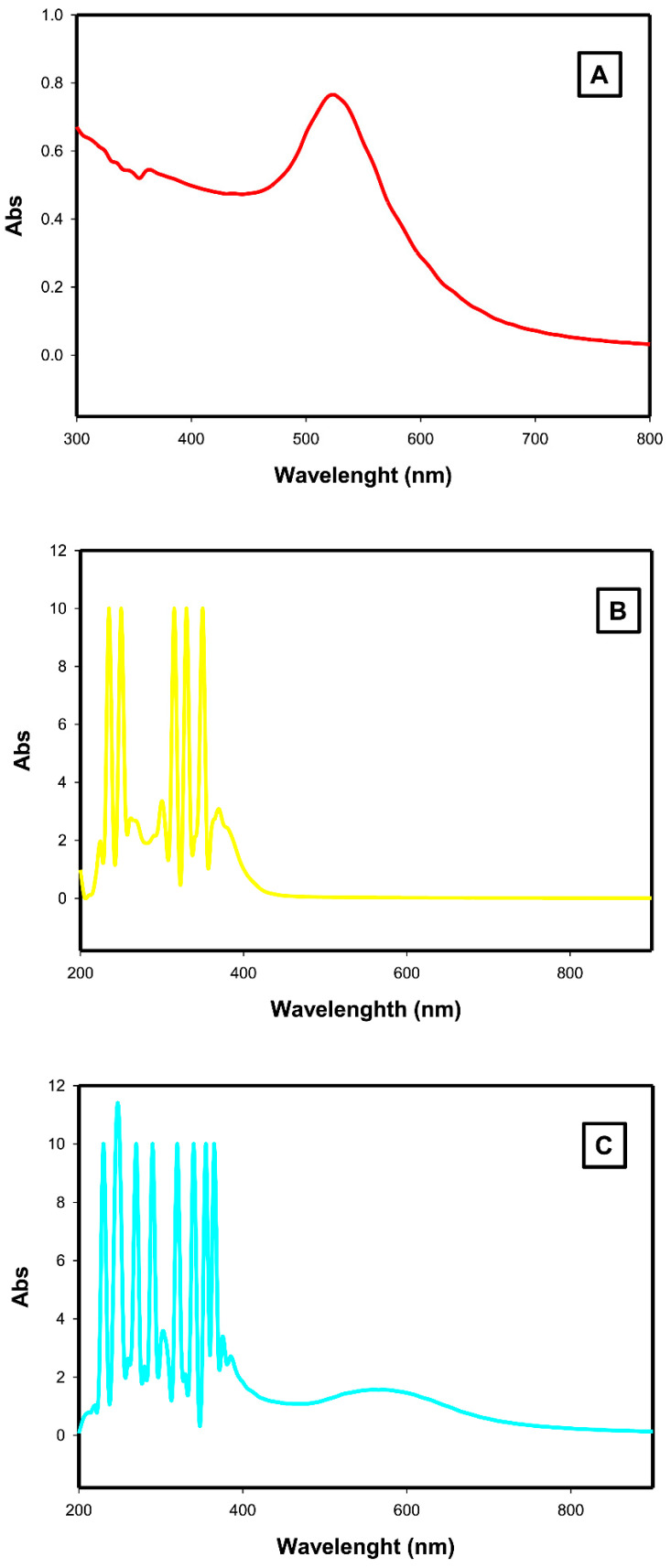
UV-visible spectra of (**A**) AuNPs, (**B**) ALA, and (**C**) AuNPs-ALA mixture.

As shown in Figure 1, AuNPs had a 520 nm specific absorption peak (Figure 1A), while ALA had a 330 nm specific absorption peak (Figure 1B). The AuNPs-ALA mixture exhibits two peaks at 560 nm (specific absorption peak of AuNPs) and at 330 nm (specific ALA absorption peak) (Figure 1C).

#### 2.1.2. Zeta Potential Analysis

**Figure 2:**
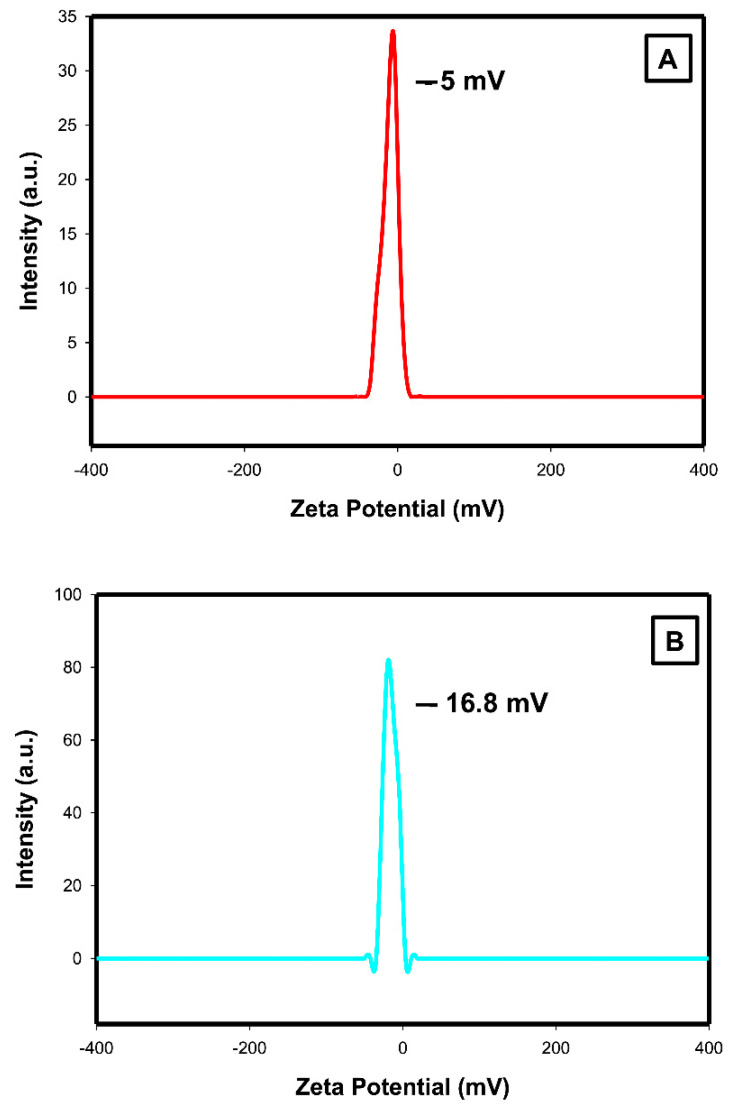
Zeta potential analysis of (**A**) AuNPs and (**B**) AuNPs-ALA.

Zeta potential of AuNPs is shown to be −5 mV (Figure 2A), whereas the AuNPs-ALA mixture exhibits higher negative Zeta potential of −16.8 mV (Figure 2B).

#### 2.1.3. X-ray Diffraction Analysis

**Figure 3:**
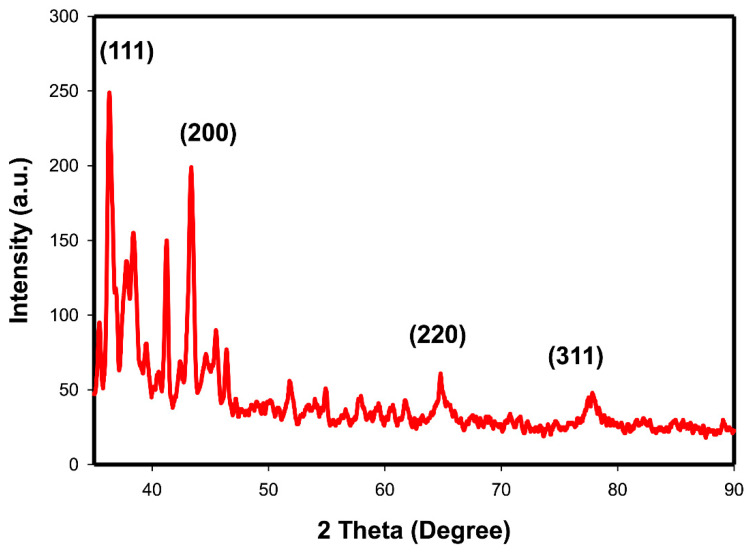
X-ray diffraction analysis of AuNPs.

As shown in Figure 3, AuNPs exhibit 4 X-ray diffraction (XRD) characteristic peaks at 2θ: 38.10°, 44.01°, 64.50°, and 77.70° corresponding to standard Bragg reflections (111), (200), (220), and (311), respectively, of face-centered cubic lattice and the intense diffraction at 38.10 peak appeared in (111) direction. These data are characteristic for AuNPs in relation to JCPDS XRD file (card no. 00-004-0784).

#### 2.1.4. Energy Dispersive Spectroscopy Analysis

**Figure 4:**
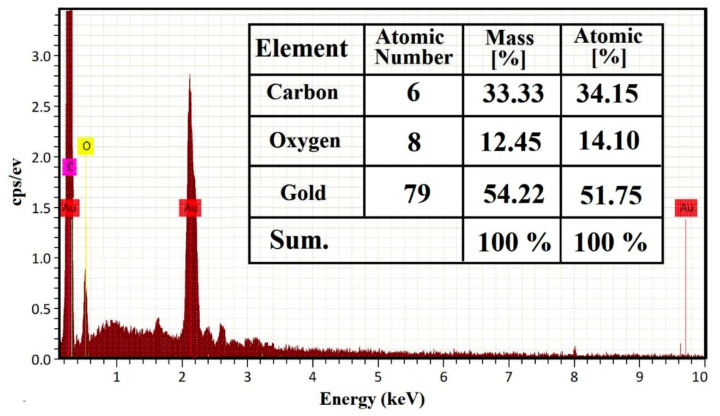
Energy dispersive spectroscopy analysis of AuNPs.

The energy dispersive spectroscopy (EDS) spectra of the AuNPs are presented in Figure 4, which reveal the existence of the Au element and carbon (C) and oxygen (O) of citrate. Au displays two major peaks of at 2.12 and 9.71 keV.

#### 2.1.5. Dynamic Light Scattering

**Figure 5:**
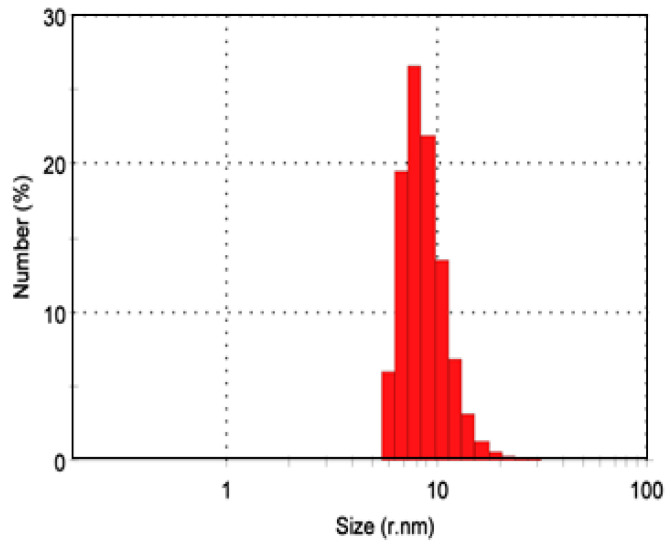
Dynamic light scattering analysis of AuNPs.

The average diameter of AuNPs is 14.85 ± 2.21 nm (Figure 5).

#### 2.1.6. Transmission Electron Microscopy

**Figure 6:**
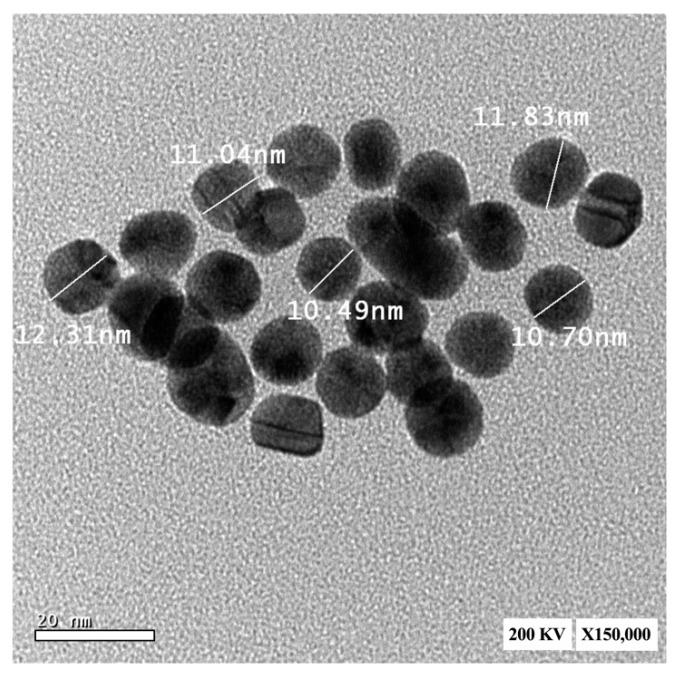
Transmission electron microscopic image of AuNPs.

As shown in Figure 6, the micrograph shows spherical monodispersed AuNPs with a mean particle size of 11.27 ± 0.34 nm.

#### 2.1.7. Fourier Transform Infrared Spectroscopy

**Figure 7:**
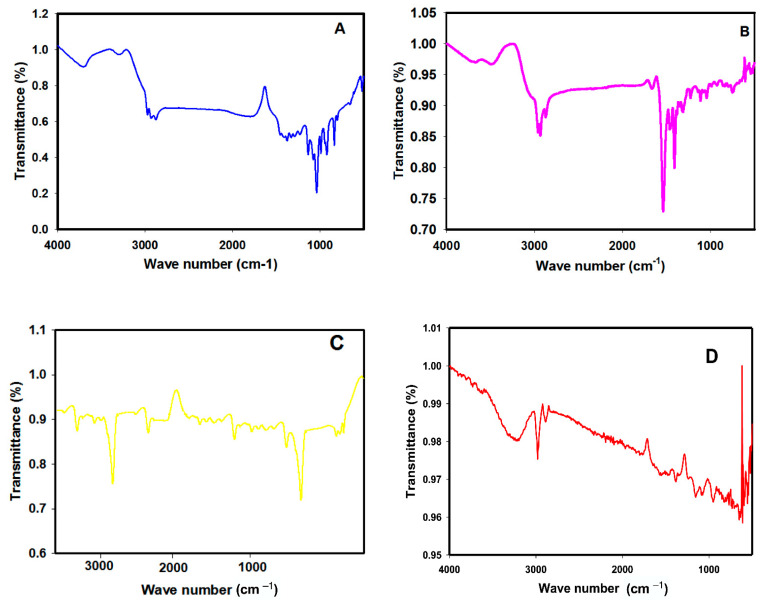

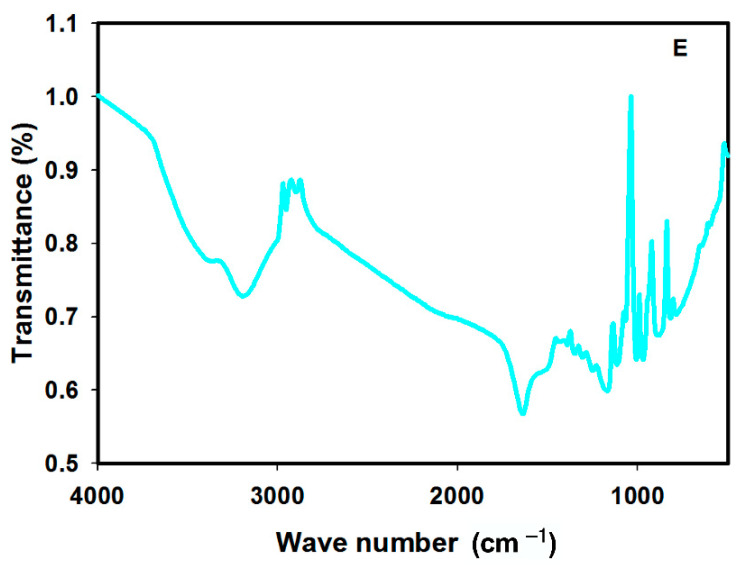
Fourier transform infrared spectroscopic spectra of (**A**) vehicle, (**B**) VPA, (**C**) ALA, (**D**) AuNPs, and (**E**) AuNPs-ALA.

The Fourier transform infrared spectroscopy (FTIR) examination was implemented to recognize the functional groups of the vehicle, VPA, ALA, AuNPs, and AuNPs-ALA, as presented in Figure 7. The FTIR spectrum of vehicle solution (Figure 7A) shows peaks at 3298.31, 2930.48, and 1038.82 cm^−1^ identical to –OH, –CH2 and C–OH stretching, consecutively, as in propylene glycol solution. The FTIR of VPA presented in Figure 7B shows peaks at the 3400 and 2961.84cm^−1^ region, indicating the –COOH peak group and –CH2 CH3, respectively. The antisymmetric carbonyl stretching mode of VPA appears as a characteristic extreme band around 1538.33 cm^−1^ and a peak 990.08 for OH. The FTIR of ALA solution (Figure 7C) shows 4 peaks at 2966.55, 1734.47, 1484.74, and 921.59 cm^−1^ corresponding to –CH2–, C=O, CH, and –OH and, finally, a peak at 660.94 cm^−1^ corresponding to weak S-S stretching vibrations. The FTIR of AuNPs (Figure 7D) show a broad peak at 3423.241 to 3373.097 cm^−1^ corresponding to O–H stretch mode of the hydroxyl group and peaks at 2921.808, 1735 cm^−1^ corresponding to stretching mode of CH and C=O, respectively. The FTIR of AuNPs-ALA mixture (Figure 7E) shows peaks indicating ALA functionalized AuNPs: peaks at 3194.84 cm^−1^ corresponding to O–H stretch mode of the hydroxyl group, 2954.64 and 1638.87 cm^−1^ corresponding to stretching mode of CH and C=O, while the weak peak of S–S band of ALA is absent in the AuNPs-ALA mixture.

### 2.2. Results of the Preliminary Experiment

#### 2.2.1. Effect of Single Oral Administration of Different Doses of AuNPs on MDA Content in Cortical and Hippocampal Tissues of 7Gy-Irradiated Rats

As presented in Table 1, normal rats given AuNPs 500 and 1000 µg/kg had no change in malondialdehyde (MDA) content in the cerebral cortex and hippocampus when contrasted to the control group, whereas AuNPs 1500 µg/kg had an elevation in MDA content in cortical and hippocampal tissues, reaching 162.38 and 237.50%, respectively, when contrasted to the control group. When contrasted to the normal control, the irradiated group had a considerable rise in MDA content in both the cerebral cortex and the hippocampus, reaching 722.45 and 853.38%, respectively. In comparison to the IRR 7Gy group, irradiated rats treated with AuNPs at a dose of 500 g/kg showed a reduction in MDA content in the hippocampus and cerebral cortex of 31.43 and 27.67%, respectively. Similarly, irradiated rats treated with AuNPs in a dose of 1000 µg/kg showed a decrease of the MDA content in cerebral cortex and hippocampus to 56.30 and 59.65%, respectively, as compared to IRR 7Gy group. Moreover, the AuNPs 1000 µg/kg + IRR group showed a decrease of the MDA content in cerebral cortex and hippocampus to 36.27 and 44.21% as compared to the AuNPs 500 + IRR 7Gy group and to 22.89 and 9.01% as compared to the AuNPs 1500 + IRR 7Gy group. Irradiated rats treated with AuNPs in a dose of 1500 µg/kg showed a decrease of the MDA content in cerebral cortex and hippocampus to 43.32 and 55.66%, respectively, as compared to the IRR 7Gy group.

#### 2.2.2. Effect of Single Oral Administration of Different Doses of AuNPs on GPX Activity in Cortical and Hippocampal Tissues of 7Gy-Irradiated Rats

As presented in Table 1, normal rats administered AuNPs 500 µg/kg and AuNPs 1000 µg/kg exhibited normal glutathione peroxidase (GPX) activity in cerebral cortex and hippocampus, whereas the AuNPs 1500 µg/kg group led to a reduction in GPX activity to 75.82 and 79.85%, respectively, in cortical and hippocampal tissues as contrasted to normal rats. Irradiated rats showed a reduction in GPX activity to 23.85 and 24.25%, respectively, in cortical and hippocampal tissues as compared to normal rats. Irradiated rats treated with AuNPs in a dose of 500 µg/kg demonstrated a partial elevation in GPX activity in the cerebral cortex and hippocampus to 144.68 and 108.80%, so as compared to the IRR 7Gy group. Treatment of irradiated rats with AuNPs in a dose of 1000 µg/kg restored GPX activity in cortical and hippocampal tissues to 305.48 and 295.59%, respectively, as compared to irradiated group.

Moreover, the AuNPs 1000 µg/kg + IRR 7Gy group showed an elevation of GPX activity in cerebral cortex and hippocampus to 65.71 and 89.46% compared to the AuNPs 500 + IRR 7Gy group and to 35.38 and 85.55% compared to the AuNPs 1500 + IRR 7Gy group. Irradiated rats treated with AuNPs in a dose of 1500 µg/kg showed a partial increase in GPX activity in both cerebral cortex and hippocampus to 199.50 and 113.20%, respectively, as compared to IRR 7Gy group.

#### 2.2.3. Effect of Single Oral Administration of Different Doses of AuNPs on DNA Fragmentation in Cortical and Hippocampal Tissues of 7Gy-Irradiated Rats

**Figure 8:**
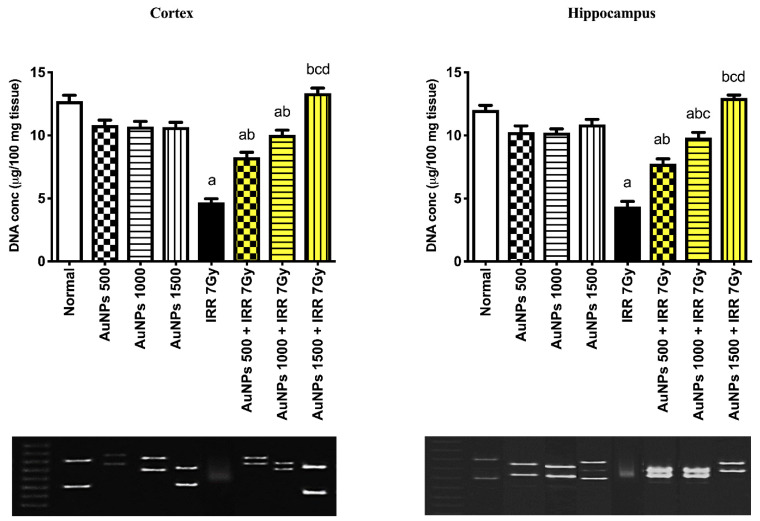
Effect of single oral administration of different doses of AuNPs on DNA in cortical and hippocampal tissues of 7Gy-irradiated rats. Every bar with a vertical line displays the mean ± S.E (*n* = 6). ^a^ vs. normal, ^b^ vs. IRR 7Gy, ^c^ vs. AuNPs 500 + IRR 7Gy, ^d^ vs. AuNPs 1000 + IRR 7G; *p* < 0.05.

As shown in Figure 8, rats receiving AuNPs at doses of 500, 1000, and 1500 µg/kg showed no DNA fragmentation in cortical and hippocampal tissues. Irradiated rats exhibited an increase in DNA fragmentation presented by a decrease in DNA content to 37.68 and 36.34% in cortical and hippocampal tissues, respectively, in comparison to the normal group. Irradiated rats that were treated with AuNPs in a dose of 500 µg/kg exhibited a partial increase in DNA concentration in both cerebral cortex and hippocampus to 75.89 and 77.43%, respectively, as compared to IRR 7Gy group. Treatment with AuNPs in a dose of 1000 µg/kg increased DNA concentration in cortical and hippocampal tissues to 113.79 and 125.15%, respectively, as it contrasted with the irradiated group. Irradiated rats treated with AuNPs in a dose of 1500 µg/kg normalized the DNA concentration in both the cerebral cortex and hippocampus.

#### 2.2.4. Effect of Single Oral Administration of Different Doses of AuNPs on Histopathological Changes in Cortical and Hippocampal Tissues of 7Gy-Irradiated Rats

**Figure 9:**
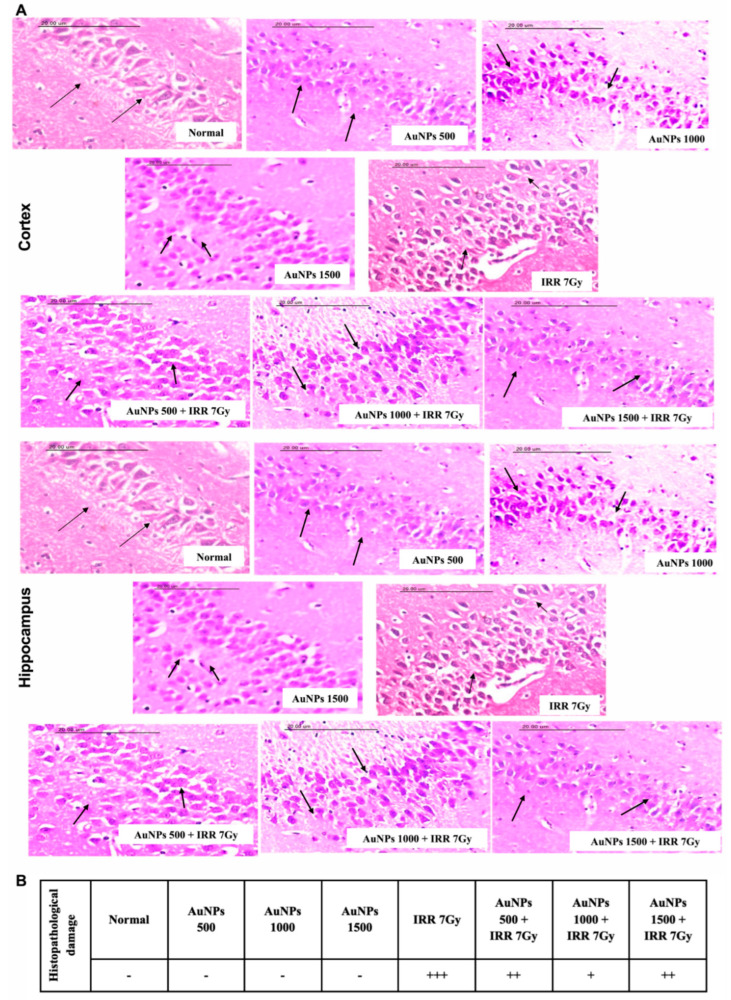
Effect of single oral administration of different doses of AuNPs on histopathological changes in cortical and hippocampal tissues of 7Gy-irradiated rats. (**A**) Sections of cortical and hippocampal specimens stained with H&E (scale bar is 20 µm). (**B**) Table showing the severity of histopathological damage.

As presented in Figure 9, histopathological examination of normal rats revealed consistent architecture for the cortical and hippocampal sections. Similarly, rats receiving AuNPs in doses of 500, 1000, and 1500 µg/kg displayed normal structure for both the cortex and hippocampus. On the other hand, cortical sections of irradiated rats showed severe damage neuronal cells’ vacuolar degeneration, focal gliosis, cerebral capillaries’ dilation with perivascular oedema, and apoptosis of neuronal cells resulted in densely basophilic structures surrounded by a halo zone. In addition, hippocampal sections revealed marked reduction of the pyramidal cell number alongside vacuolations and nuclear pyknosis of granular cell layers. Irradiated rats treated with AuNPs in a dose of 500 µg/kg showed moderate damage vacuolar degeneration of cortical neuronal cells, shrunken neuronal cells with per cellular haloes, apoptosis, gliosis, and neuronophagia, colored darkly. Furthermore, hippocampus slices exhibited cellular architecture and a reduction in the pyramidal cells’ size, as well as darker nuclei and vacuolations in the granular cell layers. In cortical sections, irradiated rats treated with AuNPs at a dose of 1000 µg/kg showed mild damage, neuronal cells’ vacuolar degeneration, gliosis, and neuronophagia, as well as cellular organization and prominent large pyramidal cells associated with darkened nuclei, and in hippocampal sections, cellular organization and prominent large pyramidal cells associated with darkened nuclei. In cortical slices of irradiated rats treated with AuNPs at a dose of 1500 µg/kg, there was moderate damage, including neuronal cells’ vacuolar degeneration, gliosis, and neuronophagia. Moreover, hippocampus slices exhibited cellular architecture as well as the preservation of tiny pyramidal cells with darker nuclei and vacuolations in the granular cell layer.

It can be concluded from the previous results that AuNPs in a dose of 1000 µg/kg showed greater effect than the other two doses in ameliorating the toxic effect of radiation on brain tissues as manifested by the significantly decreased MDA content, restored GPX activity, decreased DNA fragmentation, and increased DNA content along with showing only mild histopathological radiation-induced damage.

### 2.3. Results of the Main Experiment

#### 2.3.1. Effect of AuNPs-ALA Mixture’s Single Oral Administration on MDA Content in Cortical and Hippocampal Tissues of 7Gy-Irradiated Rats

As presented in Table 2, treatment of normal rats with VPA 200 mg/kg, ALA 100 mg/kg, AuNPs 1000 µg/kg, and AuNPs-ALA mixture groups showed no change in MDA content in both cerebral cortex and hippocampus as contrasted to the vehicle control group. In contrast, irradiation of normal rats with 7Gy caused an elevation in the MDA content in cerebral cortex and hippocampus to 645.38 and 756.97%, consecutively, as contrasted to the vehicle group. Treating irradiated rats with VPA, ALA, and AuNPs-ALA normalized the MDA content in both cortical and hippocampal tissues as compared to irradiated group. Whereas AuNPs in a dose of 1000 µg/kg partially decreased the MDA content in both cerebral cortex and hippocampus to 56.30 and 59.65%, respectively, as compared to the IRR 7Gy group.

#### 2.3.2. Effect of AuNPs-ALA Mixture’s Single Oral Administration on GPX Activity in Cortical and Hippocampal Tissues of 7Gy-Irradiated Rats

As presented in Table 2, treating normal rats with VPA 200 mg/kg, ALA 100 mg/kg and AuNPs 1000 µg/kg groups showed no change in GPX activity in hippocampus as contrasted to the vehicle control group. However, VPA, ALA, and AuNPs 1000 µg/kg groups indicated an elevation in GPX activity in cortex to 146.46, 148.00, and 148.51%, consecutively, when contrasted to the vehicle control. The AuNPs-ALA group showed no significant increase in GPX activity in both cortical and hippocampal tissues as contrasted to the vehicle control. The irradiated group exhibited a decrease in GPX activity in cortical and hippocampal tissues to 34.36 and 26.421%, consecutively, as contrasted to the vehicle group. Irradiated rats treated with VPA, ALA, and AuNPs 1000 µg/kg and AuNPs-ALA mixture restored GPX activity and ameliorated radiation damage in both cortical and hippocampal tissues, respectively, as compared to the irradiated group. Treating irradiated rats with the AuNPs-ALA mixture increased GPX activity in cortical and hippocampal tissues to 185.38 and 316.13%, respectively, as compared to IRR 7Gy.

#### 2.3.3. Effect of AuNPs-ALA Mixture’s Single Oral Administration on DNA Fragmentation in Cortical and Hippocampal Tissues of 7Gy-Irradiated Rats

**Figure 10:**
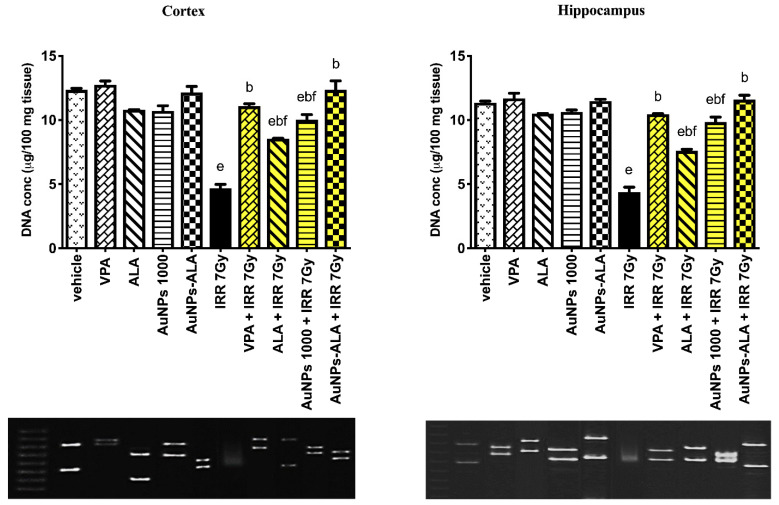
Effect of single oral administration of AuNPs-ALA mixture on DNA fragmentation in cortical and hippocampal tissues of 7Gy-irradiated rats. Every bar with a vertical line displays the mean ± S.E (*n* = 6). ^e^ vs. vehicle, ^b^ vs. IRR 7Gy, ^f^ vs. AuNPs-ALA + IRR 7Gy; *p* < 0.05.

As presented in Figure 10, VPA 200 mg/kg, ALA100 mg/kg, AuNPs 1000 µg/kg, and AuNPs-ALA mixture groups showed normal DNA concentration in the cortex and hippocampus as contrasted to the vehicle control. In contrast, the irradiated group demonstrated a reduction in DNA concentration in cortical and hippocampal tissues to 38.05 and 38.49%, consecutively, as contrasted to the vehicle control group, indicating DNA fragmentation. The VPA + IRR 7Gy group restored DNA concentration in cortical and hippocampal tissues to 135.73 and 139.15%, consecutively, as contrasted to the irradiated group. The ALA + IRR 7Gy group showed a partial increase in DNA concentration to 82.01 and 73.74% in cortical and hippocampal tissues, consecutively, as contrasted to the irradiated group. Similarly, the AuNPs 1000 + IRR 7Gy group displayed a partial increase in DNA concentration by 113.58 and 125.15% in cortical and hippocampal tissues, consecutively, as contrasted to the irradiated group. The AuNPs-ALA + IRR 7Gy group restored DNA concentration and increased DNA in both cortical and hippocampal tissues to 166.18 and 165.27%, respectively, as compared to the irradiated group.

#### 2.3.4. Effect of Single Oral Administration of AuNPs-ALA Mixture on Histopathological Changes in Cortical and Hippocampal Tissues of 7Gy-Irradiated Rats

**Figure 11:**
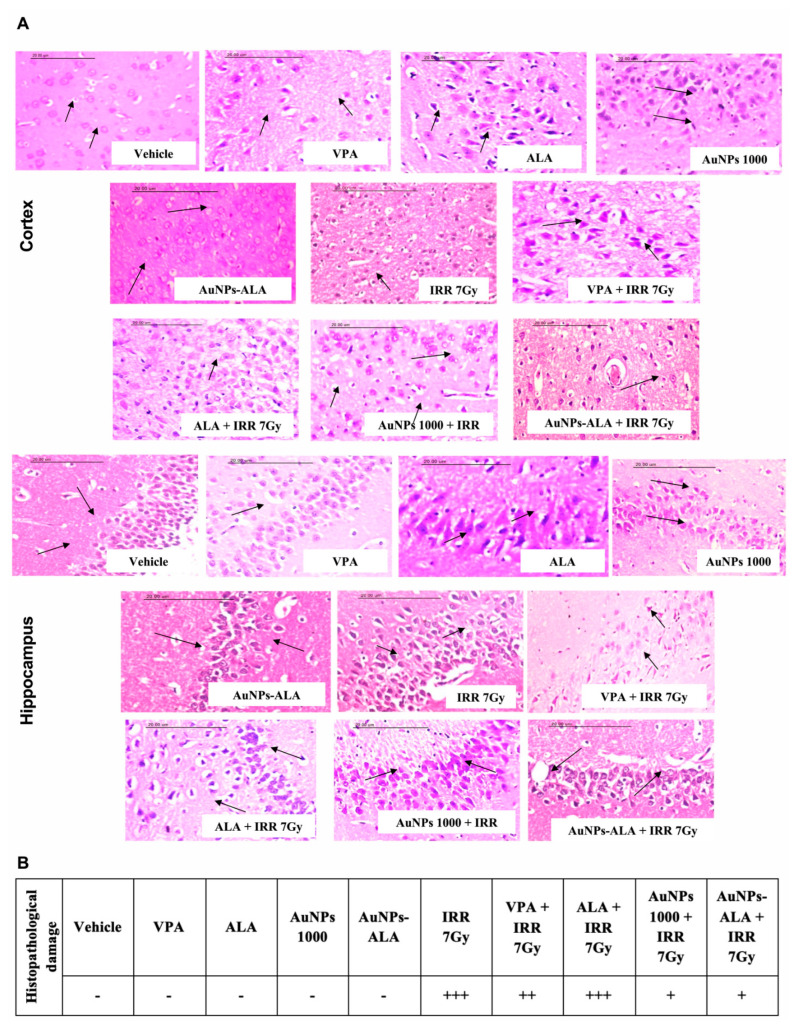
Effect of single oral administration of AuNPs-ALA mixture on histopathological changes in cortical and hippocampal tissues of 7Gy-irradiated rats. (**A**) Sections of cortical and hippocampal specimens stained with H&E (scale bar is 20 µm). (**B**) Table showing the severity of histopathological damage.

As presented in Figure 11, histopathological examination of control rats revealed consistent architecture for the cortical and hippocampal sections. Similarly, rats receiving VPA 200 mg/kg, ALA 100 mg/kg, AuNPs 1000 µg/kg, and AuNPs-ALA mixture displayed normal histological structure for both the cortex and hippocampus. On the other hand, cortical sections of irradiated rats showed neuronal cells’ vacuolar degeneration, focal gliosis, cerebral capillaries’ dilatation with perivascular oedema, massive gliosis, neuronophagia, and neuronal cells’ apoptosis which was noticed as densely basophilic bodies surrounded by a halo zone. In addition, hippocampal sections revealed marked reduction of the pyramidal cells’ number, marked vacuolations in the granular cell layers, and nuclear pyknosis which was noticed as dense basophilic bodies surrounded by a halo zone. Irradiated rats treated with VPA showed moderate damage as manifested by neuronal cells’ mild vacuolar degeneration and some darkly stained shrunken neuronal cells with per cellular haloes in cortical sections along with cellular disorganization, shrinkage in large pyramidal cells, and vacuolations in granular cell layers in hippocampal sections. Irradiated rats treated with ALA showed severe damage as manifested by neuronal vacuolar degeneration, focal gliosis, and cerebral capillaries dilatation in cortical sections along with cellular disorganization, marked shrinkage in large pyramidal cells with darkened nuclei, and marked vacuolations in granular cell layers in hippocampal sections. Irradiated rats treated with AuNPs at a dose of 1000 µg/kg showed mild damage as manifested by mild neuronal vacuolar degeneration, gliosis, and neuronophagia in cortical sections as well as cellular organization, prominent large pyramidal cells with darkened nuclei, and no vacuolations in granular cell layers in hippocampal sections. Similarly, irradiated rats treated with the AuNPs-ALA mixture showed mild damage as manifested by neuronal cells’ mild swelling, congestion, and perivascular oedema in cortical sections as well as cellular organization, mild shrinkage in pyramidal cells with darkened nuclei, and few vacuolations in granular cell layers in hippocampal sections.

## 3. Discussion

The study aim is to evaluate the potential neuroprotective impact of the AuNPs-ALA mixture against the damage induced by gamma irradiation in cortical and hippocampal tissues in rats. This study started with the synthesis and characterization of AuNPs which was then mixed with ALA to prepare the AuNPs-ALA mixture. AuNPs have a characteristic optical absorption spectrum in the UV-visible region due to surface plasmon resonance phenomena [21].

Herein, the synthesized AuNPs demonstrated a particular absorption peak at 520 nm as reported previously [22,23]. Furthermore, ALA exhibited a specific peak at 330 nm in line with GotI et al.’s work [24]. The AuNPs-ALA mixture exhibited two peaks at 560 nm, the specific absorption peak of AuNPs, and at 330 nm, the specific ALA absorption peak, which means that gold nanoparticles exhibited a bathochromic shift of 40 nm due to formation of AuNPs-ALA in accordance with Amarnath et al. who showed a bathochromic shifting of the peak of gold nanoparticles after capping with lipoic acid to (560–620 nm) [25]. Moreover, Toulbe et al. showed that the UV-VIS spectra of Au nanoparticles with sizes of 5 nm and 10 nm were situated at 522 nm and 524 nm. ALA that has interacted with Au with sizes of 5 and 10 nm, the UV-VIS spectra were characterized by absorption bands with a maximum at 322 nm and 542 nm. In the case of ALA that has interacted with Au nanoparticles with a size of 10 nm, these bands were situated at 320 nm and 530 nm in the case of ALA that has interacted with Au nanoparticles with a size of 5 nm [26]. Toulbe et al. suggested the higher affinity of thiol groups of ALA for silver (Ag) nanoparticles compared with that for Au nanoparticles [26]. The formation of the thiolate-gold clusters is explained by several models reported by Walter et al. [27] who considered the magic numbers of free valence electrons and Cheng et al. [28] who considered the super-atom network and the super-valence bond mode preferential chemical adsorption of ALA onto metallic nanoparticles, as reported in the case of other thiols [29]. The presence of Raman lines can be explained by considering that the chemical mechanism involves a charge transfer at the metal/dielectric interface the Ag or Au nanoparticles and ALA, when new bonds between the metal and adsorbate of the type Ag-S or Au-S are generated. However, Turcu et al. found that the plasmon bands in the UV-Vis spectra were bathochromic by only 10 nm owing to AuNPs-ALA functionalization [30]. In addition, Neshastehriz et al. found that bathochromic shifting confirmed the attachment of folic acid to the gold nanoparticles, and the shift in the gold nanoparticles’ UV peak in the AuNPs-ALA mixture spectrum can be attributed to formation of Au-S [23]. In addition, Chandraker et al. demonstrated that ALA capped Ag nanoparticles and suggested that ALA stabilized by Ag-S covalent interaction [31]. This behavior might be due to cross-linking. In addition, the optical behavior was pH-dependent, demonstrating that most of the ions are present in its (COO- as well as –S-) ionic state at pH greater than pKa, resulting in electrostatic stabilization of AgNPs that is more pronounced at higher pH due to disulfide bond breaking of ALA; then, two free –S groups were produced. ALA molecule capped AgNPs by covalent interaction between S-Ag. Concentration-dependent representative sets of the SPR band of TA@AgNPs show that the intensity of the SPR band was found to be inversely proportional to the concentration of TA. The shift towards longer wavelength can be attributed to the cross-linking phenomenon that was more pronounced at higher concentration. Similar results were also observed for LCys@ AgNPs and GSH@AgNPs, indicating that the AgNPs were aggregating and may be attributed to the formation of Ag–S bond and cross-linking between the adjacent groups.

The surface charges of nanoparticles play a crucial role in cell interaction; apart from size and concentration, surface charges are equally crucial to proving nanoparticle activity and stability. One of the common disadvantages of AuNPs is that they aggregated due to the Van-der-Waals force interactions between Au atoms [32]. These interactions can be minimized using a colloidal chemistry approach [33]. Zeta potential of AuNPs and AuNPs-ALA estimated in the present study were found to be −5 mV and −16.8 mV, respectively, indicating that that the mixture has a higher negative zeta potential than AuNPs alone. Sharma et al. revealed that the higher negative charge on the surface imparts higher stability owing to the electrostatic repulsion between neighboring particles [34]. AuNPs can be easily displaced, and the zeta potential is highly responsive to other molecules; Au can bind molecules by taking advantage of extremely stable thiol-gold bonds. Zeta potential in the present study confirmed the highest stability achieved with the AuNPs functionalization. In addition, the surface of AuNPs can be easily linked with different types of ligands to ensure the effectiveness of the nanoparticles, surface modifications with biomolecules, and surface chemistry improve permeability in cells/tissues, thus increase treatments and diagnosis [35].

In the present study, the crystallinity of the synthesized AuNPs and their XRD patterns were observed. Results of the XRD diffraction analysis of AuNPs revealed 4 characteristic peaks of AuNPs at 2θ going with the JCPDS XRD file (card no. 00-004-0784) as previously reported [36,37]. These 4 distinct peaks were corresponding to standard Bragg reflections (111), (200), (220), and (311) of the face-centered cubic lattice and the intense diffraction at 38.1 peak fixed in the (111) direction. This XRD pattern is typical of pure Au nanocrystals as previously reported [38]. Moreover, EDS analysis of the AuNPs herein supported the existence and formation of AuNPs due to the presence of Au metal the 2 peaks showed and the appearance of Carbon and oxygen due to citrate presence as the elemental composition of gold nanoparticles, which agree with previous work [39,40]. Collectively, EDS and XRD analyses confirmed the presence of Au metal in the AuNPs, C and O appeared in the composition of citrate in the EDS analysis; also, citrate C and O appeared as a noise in the XRD figure.

In the current study, the particle size of AuNPs determined by dynamic light scattering (DLS) was found to be ~15 nm. Moreover, AuNPs appeared as a spherical monodispersed shape with a particle size of ~12 nm using TEM examination. El-Batal et al. declared that the important biomedical applications of AuNPs originate from their size, versatility, and usability [31]. Because DLS analysis quantifies the hydrodynamic radius, the particle size acquired from DLS measurements is noticeably bigger than that acquired from TEM [31].

It is very important to determine functional groups in the examined compounds using FTIR and to characterize gold nanoparticles and gold nanoparticle conjugated systems as reported previously. Herein, the FTIR spectrum of AuNPs demonstrated peaks reported formerly [23]. Peaks in FTIR spectrum of ALA were also reported earlier [41]; however, they have not demonstrated the C–S and S–S bands. The FTIR spectrum of the present study validated ALA attachment to AuNPs as the weak peak of the S–band of ALA is absent in the AuNPs-ALA mixture and the spectral changes can be attributed to the deprotonation and formation of Au–S as previously reported by Chandraker et al. who examined the FTIR spectrum of thiotic acid and Ag nanoparticles’ weak S–S band is absent in TA@AgNPs [31]. These spectral changes can be attributed to the deprotonation and formation of Ag–S. Similar spectral changes were also observed for pure LCys, GSH and their corresponding AgNPs. In addition, the weak S–S band is absent in AgNPs-ALA which is attributed to protonation and formation of the Ag–S bond indicated successful functionalization of ALA on AgNPs’ surface [42]. The FTIR peaks of AuNPs-ALA in accordance with the IR spectrum of ALA-functionalized AuNPs showed carboxylic COOH group at 1650, 3000–3500 [30]. In addition, the FTIR of AuNPs-ALA was in accordance with Tran who investigated the synthesis and properties of ALA-stabilized gold nanoclusters [43]. In addition, the vehicle demonstrated peaks characteristic for propylene glycol solution as previously observed [44] and the VPA solution showed peaks as previously reported [45].

Direct effects of radiation initiate a chain of reactions, leading to free radicals’ formation [46]. When compared to other tissues, brain tissues are extremely vulnerable to oxidative damage [47]. The brain’s oxidative vulnerability is most likely related to its membrane phospholipids, which are high in polyunsaturated fatty acids. They are a source of peroxidation, which occurs when there is a high demand for oxygen and a lack of enzymes to clean up ROS, resulting in functional changes in nucleic acids, lipids, and proteins, which cause neurodegenerative damage. In the present preliminary study, three different doses of AuNPs 500, 1000, and 1500 µg/kg were used to record the neuroprotective dose of AuNPs against radiation caused brain damage.

Herein, the 7Gy-irradiated rats showed increased MDA content and decreased GPX activity in both brain cortex and hippocampus. These outcomes were in line with prior studies that showed an increase in lipid peroxidation after gamma radiation exposure in doses of 6Gy and 8Gy [48,49]. In addition, significant depletion of GPX activity was noticed in the brain tissues after gamma irradiation [50]. The severe damage of radiation could be explained by the membrane lipids’ peroxidation, leading to elevated membrane rigidity, reduced activity of membrane bound enzymes as well as changed permeability [51].

Results from the preliminary study revealed that usage of the three doses of AuNPs caused a significant decrease of the MDA content along with an elevation in GPX activity in the hippocampus and cortex of irradiated rats. However, the effects of the 1000 µg/kg dose were more prominent than those exerted by AuNPs 500 and 1500 µg/kg. AuNPs 1000 µg/kg restored the GPX activity and decreased the MDA content as compared to the irradiated group. This demonstrated that AuNPs at a dose of 1000 µg/kg are superior in ameliorating radiation-induced brain oxidative stress. In agreement, AuNPs in doses of 250, 500, and 1000 µg/kg alleviated the brain oxidative impairment caused by Schistosoma mansoni infection in mice as manifested by increased glutathione and decreased MDA and nitric oxide levels [52]. In addition, AuNPs ameliorated the impact of hyperglycemia induced in diabetic mice by suppressing ROS generation, scavenging free radicals, and increasing antioxidant enzymes [14]. New aproaches for nanoparticles addressed the maternal and fetal complications due to infection, mechanisms of vertical transmission, and reaction of uterine-placental innate immune [53]. Aengenheister et al. claimed that Placental uptake and translocation depend on nanoparticles’ properties as they are promising drug carriers to reduce or enhance fetal exposure [54]. In addition, it has been reported that PEGylated AuNPs (10–30 nm) revealed no placental transfer AuNPs detected in placental tissue; mainly in the ST and CT layer, not in the endothelium of fetal capillaries [55]. Moreover, AuNPs (20 nm) prevented the brain damage in okadaic acid-induced Alzheimer’s disease in rats via its anti-inflammatory and antioxidant characteristics [56]. Interestingly, it has been proposed that AuNPs are promising agents to treat neurodegenerative disorders via their antioxidant properties and easy crossing of the BBB [57]. AuNPs’ size is also critical for their toxicity. In fact, ultra-small AuNPs (1.5 nm in diameter) were found to be highly cytotoxic, while approximately 10-fold larger AuNPs (15 nm and above) were non-toxic at the same concentration levels [58]. Thus, although there is an inverse proportion between AuNPs’ size and BBB penetration, there is not a similar correlation between size and toxicity.

In the preliminary experiment, irradiated rats showed a reduction in the DNA content, indicating severe DNA fragmentation. Ionizing radiation induces ROS that can cause single- and/or double-strand breakage of DNA leading to its damage. Moreover, radiation can cause parenchymal and vascular cell death along with inhibiting the formation of new neurons in the hippocampus, ultimately leading to brain damage [59]. Herein, treatment of irradiated rats with AuNPs led to a dose-dependent elevation in the DNA content in cortical and hippocampal tissues. Noteworthy, there are competing findings in the literature concerning the effect of AuNPs on DNA damage [60], which may be attributed to the particle size of AuNPs [58]. Ultra-small particles can perfectly fit into the major grooves of the DNA, thereby immobilizing it [61,62].

Herein, the different doses of AuNPs alleviated the histopathological damage induced by 7Gy irradiation in both cortical and hippocampal tissues. Irradiated rats treated with AuNPs at doses of 500 and 1500 µg/kg showed moderate histopathological alterations in cortical and hippocampal tissues when compared to the control rats. Whereas AuNPs at a dose of 1000 µg/kg exhibited more protective effect, where irradiated rats showed only mild histopathological alterations. In accordance, AuNPs mitigated the neuronal damage in hippocampus of the amyloid β-treated rats’ model of Alzheimer’s disease [63]. Moreover, fabricated AuNPs (20 nm) ameliorated the retinal histopathological findings in streptozotocin-induced diabetic rats along with reducing retinal inflammatory cytokines, chemokines, and pro-angiogenic factors [64].

Based on the results of the preliminary study, AuNPs in a dose of 1000 µg/kg exhibited the highest protective effect against radiation-induced brain damage among the other doses. Thus, AuNPs 1000 µg/kg was selected to further examine the potential neuroprotective impact of the AuNPs-ALA mixture against the brain damage caused in 7Gy-irradiated rats.

In the main experiment, treatment of irradiated rats with the AuNPs-ALA mixture, ALA, and VPA normalized the MDA content in both cortical and hippocampal tissues, whereas AuNPs partially reduced this content. In addition, the AuNPs-ALA mixture, AuNPs, ALA, and VPA normalized also GPX activity in cortical and hippocampal tissues of irradiated rats. In accordance, Ghanizadeh showed that that AuNPs and ALA may reduce oxidative stress and neuro-inflammation in experimental models of autism [65]. This mixture showed stronger effect than that of AuNPs alone. AuNPs mixture with ALA and epigallocatechin gallate accelerated cutaneous wound healing in BALB/c mice via antioxidant activity as manifested by increased superoxide dismutase activity [66]. ALA is extremely efficient in decreasing free radicals and lipid peroxidation in different brain regions of lipopolysaccharide and polymicrobial septic rats [67,68]. Moreover, ALA increased the cerebral glutathione level and GPX activity in hippocampus of the pilocarpine-induced seizure model in rats [69].

In the present findings, the AuNPs-ALA mixture and VPA normalized the DNA content in both cortical and hippocampal tissues of irradiated rats, whereas AuNPs and ALA partially increased the DNA content. The AuNPs-ALA mixture showed superior effect compared to that of AuNPs or ALA alone. ALA treatment reduced ethanol-induced DNA damage in the developing hippocampus and cerebellum of rats [70]. In addition, ALA reduced the DNA fragmentation after spinal cord injury in rats through its anti-inflammatory and antioxidant properties [71]. ALA combats oxidative stress and protects against DNA damage caused by peroxy and nitroso radicals [72]. These impacts are thought to be owing to ALA’s antioxidant properties, which are mediated via antioxidant enzymes’ reactivation or free radical generation suppression [20]. Furthermore, ALA induced a significant elevation in nucleic acid and protein content in elderly rats, which could be due to the lipoate’s ability to reverse poly-ADP-ribosylation-associated DNA damage [73]. Previous studies such as those of Choi et al. reported that ALA can cross BBB as well and it is well known with its neurorestorative effect [74].

In the present study, the AuNPs-ALA mixture and AuNPs alleviated the histopathological damage caused by 7Gy irradiation in both cortical and hippocampal tissues, as manifested by the observed mild histopathological alterations when compared to control rats. Whereas VPA and ALA-treated groups showed moderate and severe histopathological damage, respectively. Though, it was previously reported that ALA pretreatment reduced radiation-induced cerebellar neuronal damage [75], the inability of ALA herein to ameliorate the neuronal damage may be due to the short duration of ALA treatment. Results of the main experiments revealed that the AuNPs-ALA mixture showed superior effect compared to that of AuNPs or ALA alone in terms of alleviating oxidative stress, DNA damage, and histopathological injury collectively.

## 4. Materials and Methods

### 4.1. Bioethical Statement

The National Center for Radiation and Technology (NCRRT) in Cairo, Egypt, provided male Wistar albino rats weighing 150–180 g. Rats were kept in standard laboratory conditions of temperature (25 ± 2 °C), relative humidity (60–70%), and a 12 h light cycle throughout the study. They were given ad libitum water and standard pellet chow (El-Nasr Chemical Co., Cairo, Egypt). The experimental protocol (Permit Number: PT1831) was authorized by the Faculty of Pharmacy’s Ethics Committee for Animal Experimentation in Cairo, Egypt. Methods were performed according to the Laboratory Animal Care and Use Guide (NIH Publications No. 85-23, revised 2011).

### 4.2. Chemicals and Drugs

Gold (III) chloride hydrate (HauCl4), ALA, VPA, trisodium citrate dehydrate, and propylene glycol were acquired from Advent Chembio PVT. LTD. (Navi Mumbai, Maharashtra, India), EVA Pharma (Cairo, Egypt), Sanofi Egypt (Cairo, Egypt), Sigma-Aldrich (St. Louis, MI, USA), and El Gomhoreya Co. (Cairo, Egypt), respectively. All other chemicals were of the greatest analytical level.

### 4.3. Preparation of Gold Nanoparticles

Gold nanoparticles (~12 nm diameter) were synthesized as mentioned previously using the citrate reduction method of HauCl4 [76,77] in the Drug Radiation Research Laboratory, NCRRT, Cairo, Egypt. Briefly, 1 mM solution of HauCl4 (5 mg/mL) was mixed with a solution of Na citrate prepared in deionized water (the molar ratio of citrate to gold is 5/1). The reaction was performed in a dark environment at 37 °C. After 1 h, it was observed that the color of the solutions turned to dark purple from light yellow. The prepared AuNPs solution had a concentration of 200 µg/mL doses of 500, 1000, and 1500 µg/kg that were equivalent to 0.3125, 0.625 mL, and 0.9375 mL, respectively. Finally, characterization of AuNPs was performed using different techniques.

### 4.4. Preparation of Drugs’ Solutions

Alpha-lipoic acid is sparingly soluble in water; therefore, it was solvated in a 50% *v*/*v* mixture from deionized water (PH = 8.5) and propylene glycol as previously described [78]. The selected oral dose of ALA was 100 mg/kg based on the existing literature [20,73]. ALA solution was prepared with a final concentration of 20 mg/mL. The dose of 100 mg/kg was equivalent to 0.625 mL for each rat. Whereas VPA was dissolved in distilled water and given to rats at a dose of 200 mg/kg orally [79]. The AuNPs-ALA mixture was freshly prepared by mixing equal volumes of AuNPs and ALA as suggested previously [66]. AuNPs 1000 µg/kg (0.625 mL) and ALA 100 mg/kg (0.625 mL) solutions were added in a test tube to yield 1.25 mL of mixture to be orally administered to rats. The vehicle solution was synthesized by the same method for the mixture synthesis without the addition of both AuNPs and ALA.

In the preliminary experiment, AuNPs were administered orally in 3 different doses: 500, 1000 [52], and 1500 µg/kg, to determine the neuroprotective effect of AuNPs. The dose of AuNPs 1000 µg/kg was chosen to be mixed with ALA (100 mg/kg), to produce a mixture of AuNPs-ALA.

### 4.5. Gold Nanoparticles’ Characterization

#### 4.5.1. Ultraviolet/Visible Spectroscopy

The absorption spectrum of AuNPs was detected by a double beam UV/VIS spectrophotometer (JASCO V-560 UV-VIS, Shimadzu, Japan) within a range between 200–900 nm, a 1 nm resolution, and a 0.3 nm/s-scan rate using bi-distilled water as a blank [80]. Similarly, ALA and AuNPs-ALA were identified in the prepared solutions using the vehicle solution as a blank.

#### 4.5.2. Zeta Potential Analysis

Zeta potential determines the solutions’ stability and surface charge, which could enhance its adsorption capability [81]. Zeta potential and surface charge in the present study of AuNPs and the AuNPs-ALA mixture is estimated using the Malvern device, UK.

#### 4.5.3. X-ray Diffraction Analysis

X-ray diffraction technique was used to determine the crystalline nature of the synthesized NPs and their XRD patterns. XRD analysis was estimated in the present study using XRD-6000 (Shimadzu scientific instruments, Kyoto City, Japan), and utilized a Cu-Ka and nickel filter. Working with a Cu current at 30.0 mA at a voltage 40.0 kV, the intensity of the diffracted X-rays was evaluated as a function of the diffracted angle 2θ [82].

#### 4.5.4. Energy Dispersive Spectroscopy

The EDS was used to determine the elemental composition, purity, and the distribution of elements in the prepared samples. The EDS analysis of AuNPs was employed using EDX BRUKER, Nano GmbH, D-12489, 410-M equipment, Germany [82].

#### 4.5.5. Dynamic Light Scattering

Hydrodynamic diameter was estimated by the PSS-NICOMP 380-ZLS particle sizing system (Santa Barbara, CA, USA) based on the DLS technique [80].

#### 4.5.6. Transmission Electron Microscopy

The shape and particle size of AuNPs were examined utilizing a 200 kV JEOL JEM-2100 transmission electron microscope (TEM; Tokyo, Japan) [80]. Image analysis software was then utilized to determine the particle size and size distribution.

#### 4.5.7. Fourier Transform Infrared Spectroscopy

The FTIR method was conducted to identify the functional groups available in AuNPs, ALA, AuNPs-ALA mixture, and VPA solutions using a JASCO FT-IR-3600 infra-red spectrometer (Tokyo, Japan) within a wavelength ranging from 400 to 4000 cm^−1^ at a resolution of 4 cm^−1^.

### 4.6. Irradiation of Animals

The irradiation was done at NCRRT, Egyptian Atomic Energy Authority, Cairo, Egypt, using an AECL Gamma cell − 40 biological indicator. An acute single dose of 7Gy was given to rats at a dose rate of 0.704 rad/sec [50].

### 4.7. Experimental Design

The current study included two experiments. The first one was the preliminary experiment that was conducted to determine the possible neuroprotective dose of AuNPs in the gamma-irradiated rats. Three doses of AuNPs were examined according to the previous study of Dkhil et al. [52] who assessed the neuroprotective effect of AuNPs in the brain against Schistosoma mansoni infection using three different concentrations: 0.25, 0.5, and 1 mg/kg. Similarly, in the current work, the doses 500 and 1000 µg/kg were used in addition to the dose of 1500 µg/kg, to determine the optimal neuroprotective dose against radiation-induced damage in rats’ cortex and hippocampus.

Sixty-four rats were randomly allocated between eight groups (*n* = 8) as follows:

Group I: rats received a single oral dose of saline.
Group II: normal rats were irradiated with a single dose of 7Gy gamma radiation.
Group III: normal rats get 500 µg/kg AuNPs’ single oral dose.
Group IV: normal rats get 1000 µg/kg AuNPs’ single oral dose.
Group V: normal rats get 1500 µg/kg AuNPs’ single oral dose.
Group VI: irradiated rats get 500 µg/kg AuNPs’ single oral dose.
Group VII: irradiated rats get 1000 µg/kg AuNPs’ single oral dose.
Group VIII: irradiated rats get 1500 µg/kg AuNPs’ single oral dose.

All rats were irradiated 1 h after receiving the oral dosage of AuNPs in the irradiated groups. One day following irradiation, under light anesthesia [83], the rats were sacrificed, and their brains were properly removed, washed, dried, and dissected to separate the cortex and hippocampus. Specimens from each rat’s brain (*n* = 6) were separated into two halves, weighed, and stored at −20 °C for later investigations. The first half of cortical and hippocampal tissues were homogenized in 5% *w*/*v* 20 mM phosphate buffer (pH = 7.4) and centrifuged; then, supernatants were used to measure the MDA content and GPX activity. Cortical and hippocampal tissues from the other halves were used to assess DNA fragmentation. Finally, two brain samples from each group were collected and kept in neutral buffer formalin 10% for histopathological examination

Results of the present preliminary study revealed that 1000 µg/kg was the most effective neuroprotective dose of AuNPs; thus, this dose was utilized to conduct the second experiment. AuNPs (1000 µg/kg) was mixed with ALA to form the AuNPs-ALA mixture. ALA was previously reported by several researchers as a neuroprotective drug and specifically at a dose of 100 mg/kg [20,73]. The neuroprotective effects of this mixture were assessed in the cortical and hippocampal tissues of the brain of irradiated rats.

In this experiment, eighty rats were categorized between ten groups (*n* = 8) at random as follows:

Group I: normal rats get the vehicle’s single oral dose.
Group II: normal rats get 200 mg/Kg VPA’s single oral dose.
Group III: normal rats get 100 mg/kg ALA’s single oral dose.
Group IV: normal rats get 1000 µg/kg AuNPs’ single oral dose.
Group V: normal rats get freshly prepared AuNPs-ALA mixture’s single oral dose (mixture of 100 mg/kg ALA and 1000 µg/kg AuNPs).
Group VI: normal rats irradiated with a single dose of 7Gy gamma radiation.
Group VII: irradiated rats get 200 mg/Kg VPA’s single oral dose.
Group VIII: irradiated rats get 100 mg/kg ALA’s single oral dose.
Group IX: irradiated rats get 1000 µg/kg AuNPs’ single oral dose.
Group X: irradiated rats get freshly prepared AuNPs-ALA mixture’s single oral dose.

All rats were irradiated 1 h after receiving the oral dosage of the drugs in the irradiated groups. One day following irradiation, rats were exposed to the same procedures and investigations described above in the preliminary experiment.

### 4.8. Colorimetric Assays

The MDA content and GPX activity were estimated in cortical and hippocampal tissues using specific colorimetric kits acquired from Eagle Biosciences Inc. (Catalog no: LIP39-K01, Boston, MA, USA) and BioVision Incorporated (Catalog no: K762-100, San Francisco, CA, USA), respectively, according to manufacturer’s protocol.

### 4.9. DNA Fragmentation

Cortical and hippocampal tissue homogenates were subjected to DNA extraction using the Zymoresearch Quick-gDNA™ MiniPrep kit (Catalog No: D3024, Irvine, CA, USA). Tissue homogenates were centrifuged for 10 min (12,000× *g*, 4 °C). Supernatants were utilized for DNA isolation. At the end of the extraction process, the resultant DNA was electrophoresed using tris acetate-EDTA buffer on a 2 percent agarose gel comprising ethidium bromide. Each lane was loaded with the same amount of DNA, and a molecular DNA marker was utilized as a molecular mass standard. As previously reported, the DNA was seen under UV illumination and its concentration was determined [84].

### 4.10. Histopathological Examination

Brain samples were fixed in a 10% neutral buffered formalin solution, then dehydrated in rising concentrations of ethanol, cleaned in xylene, embedded in paraffin wax, and partitioned at a thickness of 5 microns. A light microscope was used to examine sections of prepared slides stained with hematoxylin and eosin [85]. Examination of brain tissue sections was scored according to the degree of necrosis, hemorrhage, and vacuolations as follows: –, no visible abnormalities; +, mild; ++ moderate; +++, severely damaged [86].

### 4.11. Statistical Analysis

Data were indicated as means ± SE. Data were measured by one way Analysis of Variance (ANOVA) accompanied by Tukey–Kramer as post comparisons test. The difference between means was significant at *p* < 0.05.

## 5. Conclusions

Dependent on the observations of the current study, it can be obtained that the AuNPs-ALA mixture exerts central neuroprotective effects against the damage activated by gamma radiation in rats through getting rid of oxidative stress, DNA damage, and histopathological damage in both cortical and hippocampal tissues. Such effects were superior to those exerted by each component alone. Thus, the AuNPs-ALA mixture may be considered a potential candidate for addressing radiation-associated brain toxicity.

## Figures and Tables

**Table 1:** Effect of single oral administration of different doses of AuNPs on MDA content and GPX activity in cortical and hippocampal tissues of 7Gy-irradiated rats.

Groups	MDA (μM/mg Protein)		GPX (nmol/mg Protein)	
	Cortex	Hippocampus	Cortex	Hippocampus
Normal	0.561 ± 0.006	0.384 ± 0.002	2.527 ± 0.014	1.688 ± 0.022
IRR 7Gy	4.053 ± 0.166 ^a^	3.277 ± 0.198 ^a^	0.602 ± 0.037 ^a^	0.409 ± 0.067 ^a^
AuNPs 500	0.600 ± 0.013	0.492 ± 0.022	2.610 ± 0.089	1.762 ± 0.031
AuNPs 1000	0.560 ± 0.011	0.447 ± 0.0142	2.605 ± 0.079	1.728 ± 0.014
AuNPs 1500	0.911 ± 0.027 ^a^	0.912 ± 0.013 ^a^	1.916 ± 0.024 ^a^	1.348 ± 0.032 ^a^
AuNPs 500 + IRR 7Gy	2.779 ± 0.029 ^ab^	2.370 ± 0.103 ^ab^	1.473 ± 0.075 ^ab^	0.854 ± 0.0119 ^ab^
AuNPs 1000 + IRR 7Gy	1.771 ± 0.023 ^abc^	1.322 ± 0.020 ^abc^	2.441 ± 0.024 ^bc^	1.618 ± 0.024 ^bc^
AuNPs 1500 + IRR 7Gy	2.297 ± 0.021 ^abcd^	1.453 ± 0.012 ^abc^	1.803 ± 0.020 ^abd^	0.872 ± 0.020 ^abd^

Data are presented as mean ± SE (*n* = 8). ^a^ vs. normal group, ^b^ vs. IRR 7Gy group, ^c^ vs. AuNPs 500 + IRR 7Gy group, ^d^ vs. AuNPs 1000 + IRR 7Gy group at *p* < 0.05.

**Table 2:** Effect of single oral administration of AuNPs-ALA mixture on MDA content and GPX activity in cortical and hippocampal tissues of 7Gy-irradiated rats.

Groups	MDA (μM/mg Protein)		GPX (nmol/mg Protein)	
	Cortex	Hippocampus	Cortex	Hippocampus
Vehicle	0.628 ± 0.023	0.426 ± 0.05	1.754 ± 0.043	1.548 ± 0.028
VPA	0.579 ± 0.003	0.394 ± 0.007	2.569 ± 0.030 ^e^	1.756 ± 0.013
ALA	0.570 ± 0.002	0.377 ± 0.005	2.596 ± 0.025 ^e^	1.818 ± 0.024
AuNPs 1000	0.560 ± 0.011	0.447 ± 0.014	2.605 ± 0.079 ^e^	1.728 ± 0.014
AuNPs-ALA	0.642 ± 0.025	0.427 ± 0.070	1.753 ± 0.034	1.547 ± 0.079
IRR 7Gy	4.053 ± 0.166 ^e^	3.277 ± 0.198 ^e^	0.602 ± 0.037 ^e^	0.409 ± 0.067 ^e^
VPA + IRR 7Gy	0.610 ± 0.032 ^b^	0.433 ± 0.028 ^b^	3.627 ± 0.184 ^ebf^	2.978 ± 0.097 ^ebf^
ALA + IRR 7Gy	0.646 ± 0.045 ^b^	0.516 ± 0.042 ^b^	2.474 ± 0.020 ^ebf^	1.472 ± 0.055 ^b^
AuNPs 1000 + IRR 7Gy	1.771 ± 0.023 ^ebf^	1.322 ± 0.020 ^ebf^	2.441 ± 0.024 ^ebf^	1.618 ± 0.024 ^b^
AuNPs-ALA + IRR 7Gy	0.718 ± 0.025 ^b^	0.361 ± 0.035 ^b^	1.718 ± 0.018 ^b^	1.702 ± 0.039 ^b^

Data are presented as mean ± SE (*n* = 8). ^e^ vs. vehicle group, ^b^ vs. IRR 7Gy group, ^f^ vs. AuNPs-ALA + IRR 7Gy group at *p* < 0.05.

## Data Availability

Data is contained within the article.
